# Ridge Penalization in High-Dimensional Testing With Applications to Imaging Genetics

**DOI:** 10.3389/fnins.2022.836100

**Published:** 2022-03-24

**Authors:** Iris Ivy Gauran, Gui Xue, Chuansheng Chen, Hernando Ombao, Zhaoxia Yu

**Affiliations:** ^1^Biostatistics Group, Computer, Electrical, Mathematical Sciences, and Engineering Division, King Abdullah University of Science and Technology, Thuwal, Saudi Arabia; ^2^Center for Brain and Learning Science, Beijing Normal University, Beijing, China; ^3^Department of Psychological Science, University of California, Irvine, Irvine, CA, United States; ^4^Department of Statistics, University of California, Irvine, Irvine, CA, United States

**Keywords:** high-dimensional testing, genome-wide association studies, neuroimaging, ridge penalization, imaging genetics

## Abstract

High-dimensionality is ubiquitous in various scientific fields such as imaging genetics, where a deluge of functional and structural data on brain-relevant genetic polymorphisms are investigated. It is crucial to identify which genetic variations are consequential in identifying neurological features of brain connectivity compared to merely random noise. Statistical inference in high-dimensional settings poses multiple challenges involving analytical and computational complexity. A widely implemented strategy in addressing inference goals is penalized inference. In particular, the role of the ridge penalty in high-dimensional prediction and estimation has been actively studied in the past several years. This study focuses on ridge-penalized tests in high-dimensional hypothesis testing problems by proposing and examining a class of methods for choosing the optimal ridge penalty. We present our findings on strategies to improve the statistical power of ridge-penalized tests and what determines the optimal ridge penalty for hypothesis testing. The application of our work to an imaging genetics study and biological research will be presented.

## 1. Introduction

Even with the advancements of genome-wide association studies over the past two decades, unraveling the genetic basis of many complex neurological conditions remains to be a challenge. Often, each individual's genetic information has a small contribution to disease risk and can be highly heterogeneous (Peper et al., 2007; Marenco and Radulescu, 2010; Tost et al., 2012; Batmanghelich et al., 2013). Imaging genetics offers an approach to understanding the genetic basis of neurological disorders by investigating the integrated multi-scale genomic data, multimodal brain imaging information, and environmental risk factors (Thompson et al., 2013; Nathoo et al., 2019). The rationale for imaging genetics is that by examining single nucleotide polymorphisms (SNPs), we may discover essential insights into the brain-relevant genetic polymorphisms to understand better the neural architecture through which psychopathology may emerge. Typically, these studies involve a small number of subjects relative to the amount of information available per subject, such as millions of SNPs, thousands of genetic variants or differentially methylated probes, hundreds of thousands of voxels, and dozens to hundreds of electroencephalogram (EEG) channels. Hence, both explanatory and response variables in imaging genetics studies can be high-dimensional in nature.

However, the joint analysis of both high-dimensional imaging and genetic data presents major computational and theoretical challenges for existing analytical methods (Nathoo et al., 2019) as well as the proliferation of false discoveries (Meyer-Lindenberg et al., 2008). Widely-implemented methods to fit high-dimensional statistical models include penalized regression where some form of regularization is imposed. The penalized regression literature generally adopts the perspective of maximum likelihood theory. In the context of linear regression, the negative log likelihood or loss function has the form $L=||\text{Y}-\text{X}\beta |{|}_{2}^{2}$ where **X** is an *n* × *p* design matrix of explanatory variables and **Y** is an *n* × *q* matrix of responses. The classic, unique solution minimizing the loss function L is ${\hat{\beta }}_{\text{OLS}}={\left({\text{X}}^{⊤}\text{X}\right)}^{-1}{\text{X}}^{⊤}\text{Y}$ when *n* > *p* and **X** has full column rank.

As *p* increases for a fixed *n*, the direct application of regression model and likelihood-based methods are encumbered by several issues. For example, an overfitted model may lead to large variance and low performance in testing data, i.e., low generalization. When the number of explanatory variables exceeds the sample size, the least squares estimate is not unique because the computation involves inverting the singular **X**^⊤^**X**. Regularization methods are often adopted to overcome these problems, in which case the objective function is modified to be *Q*(***β***∣**X**, **Y**) = L(***β*** ∣ **X**, **Y**) + *P*_λ_(***β***) where *P*_λ_ is a penalty function and λ is a tuning parameter. For γ ≥ 0, the *L*_γ_ norm of ***β*** is formally defined as


(1) $$\begin{array}{l} ||\beta |{|}_{\gamma }={\left(\overset{p}{\underset{j=1}{\sum }}|\beta_{j}{|}^{\gamma }\right)}^{1/\gamma }. \end{array}$$

This class of well-known penalization functions and criteria aim to balance the trade-off between bias and variance or between complexity and generalization. For example, both AIC and BIC, two well-known criteria, belong to the *L*_0_ norm, as ||***β***||_0_ is the number of non-zero elements in ***β***. The *L*_1_ norm is often considered as a convex relaxation of the *L*_0_ norm, and it achieves both sparsity and computational efficiency. The *L*_2_ norm, the penalty of which is often known as the ridge penalty or Tikhonov regularization, was motivated for ill-conditioned or close to ill-conditioned problems (Tikhonov, 1943; Hoerl, 1962; Hoerl and Kennard, 1970). Ridge penalty has also been used alone or combined with *L*_1_ in high-dimensional inference problems and deep neural networks. For example, for genetic predictive problems or association studies, ridge penalty has been widely used (Hayes et al., 2001; Liu et al., 2007; Cule et al., 2011; de los Campos et al., 2013; Lin et al., 2013, 2016; Zhao and Zhu, 2019).

More recently, ridge regression has been intensively studied as a way to try to understand why overfitted models can have satisfactory predictive performance in testing data. For example, it has been observed that models trained using deep neural networks not only have an almost perfect fit to the training data, but also generalize well to testing data (Zhang et al., 2016). Recall that a ridge regression applies an *L*_2_ penalty, i.e., the corresponding objective function is


(2) $$\begin{array}{l} Q\left(\beta |\text{X},\text{Y}\right)=||\text{Y}-\text{X}\beta |{|}_{2}^{2}+\lambda ||\beta |{|}_{2}^{2}. \end{array}$$

The ridge penalty is particularly attractive to work with because the maximum penalized likelihood estimator has a simple closed form. This objective function is differentiable and it is straightforward to show that its minimum occurs at


(3) $$\begin{array}{l} {\hat{\beta }}_{\lambda }={\left({\text{X}}^{⊤}\text{X}+\lambda {\text{I}}_{p}\right)}^{-1}{\text{X}}^{⊤}\text{Y}. \end{array}$$

Thus, the ridge solution includes the ordinary least squares solutions as a special case when λ = 0.

The ridge regression methodology yields a class of biased estimators, and massive literature is driven toward identifying an optimal ridge penalty parameter to be used in practice. The primary objective is to ensure that the ridge estimator has lowest mean squared error (Hoerl and Kennard, 1970). This translates to the pragmatic goal of developing methods which produce ridge estimates that are more useful than the least squares estimates. Despite the numerous available methods for choosing an optimal value, the ultimate choice of λ for a specific application still remains unsolved because the optimal level of regularization usually depends on the unknown characteristics of the data generating distribution (Patil et al., 2021).

As ridge regression is mathematically neat and relatively easy to study, it has been recently widely used as the first attempt to understand under what circumstances overfitting is harmless or benign, especially in high-dimensional settings. A variety of combinations have been examined, such as asymptotic or fixed sample sizes, random coefficients or fixed coefficients, the ratio of *p* to *n*, and conditional on or marginalize the covariate matrix. A representative but by no means complete list of studies include (Randolph et al., 2012; Dobriban and Wager, 2018; Hastie et al., 2019; Bartlett et al., 2020; Kobak et al., 2020; Patil et al., 2021) and some of them are credited for introducing eye-catching phrases such as “benign overfitting” and “double descent”. These studies involving ridge regression are devoted to either performance of prediction or regression coefficient estimation. To the best of our knowledge, no systematic work has been conducted to investigate the role that ridge penalty plays in high-dimensional hypothesis testing.

Moreover, to explore the relationship of neurological and genetic information in imaging genetic studies, we are interested in determining whether given sets of features are significantly associated in aggregate. In this study, we will utilize one of the extensions (Pluta et al., 2021) of the classical Mantel test (Mantel, 1967) to characterize the association between two potentially high-dimensional distance matrices. The Mantel test (Mantel, 1967) is an easy-to-implement and flexible procedure, which was originally motivated by assessing the association between the temporal and spatial relationship of leukemia cases and similar diseases. As presented in Mantel (1967), the temporal-spatial association can be examined by using the correlation of the temporal and spatial distance matrices of the observed leukemia cases. Similar or modified approaches have been commonly applied, such as identifying the spatial pattern of genetic variation by correlating genetic and geographic distances (Diniz-Filho et al., 2013). In the extension presented by Pluta et al. (2021), a Mantel-type of test with ridge regularization was presented as a compromise between the score tests from fixed-effects and random-effects model. The overarching goal of this study is to examine a class of methods for choosing the optimal ridge penalty parameter and incorporate these in the Adaptive Mantel test (Pluta et al., 2021) for hypothesis testing problems with high-dimensional data set up.

Our contribution to the initial work by Pluta et al. (2021) is three-fold. First, we propose a thresholding procedure aligned to the philosophical considerations of ridge regression in high-dimensional settings. In this study, we allow the set of candidate values of λ to include negative values and investigate how these negative penalty parameters can affect the corresponding Type I error and empirical power of the Adaptive Mantel Test. Second, we extend the AdaMant algorithm to include the selection of the optimal ridge penalty parameter *via* generalized cross-validation. To illustrate the almost sure convergence results in Patil et al. (2021) using imaging genetics data, we also implement the selection of the ridge penalty parameter using leave-one-out cross-validation. The resulting optimal choice between the two cross-validation procedures will be compared. Third, we also investigate the Type I error rate and empirical power of the test using the parametric asymptotic null distribution.

This article is outlined as follows. The general frameworks of Mantel Test and score test in linear models are presented in Sections 2.1 and 2.2. Some existing procedures for selecting the ridge penalty parameter are discussed in Section 2.3. The rationale and contributions of our work are illustrated in Section 2.4. The class of methods for choosing the optimal ridge penalty are presented in Section 3. Finally, the numerical studies involving the proposed methods and the application to an imaging genetics data set are available in Section 4.

## 2. Related Work

### 2.1. Mantel Test

Suppose we have $\left({\text{X}}_{i},{\text{Y}}_{i}\right)\in {\mathbb{R}}^{p}\times {\mathbb{R}}^{q}$ for all subjects *i* = 1, 2, …, *n* where *p* is the number of explanatory variables and *q* is the number of response variables. In imaging genetics studies, the value of *p* usually correspond to the total number of genetic variations, such as single nucleotide polymorphisms (SNPs) in genomics or differentially methylated probes in epigenetics. Meanwhile, the response variables correspond to the brain imaging information, such as pairwise alpha-band coherence measures obtained from several EEG channels.

Suppose **X**_*i*_ and **X**_*j*_ correspond to the vector of explanatory variables for subjects *i* and *j*, respectively. As described in Pluta et al. (2021), let K^**X**^(·, ·) and K^**Y**^(·, ·) be positive semi-definite kernel functions on **X** × **X** and **Y** × **Y**, respectively where the data matrices **X** and **Y** are column-centered. Specifically, we are interested in investigating the kernel function $K^{\text{X}}\left({\text{X}}_{i},{\text{X}}_{j}\right)={\text{X}}_{i}^{⊤}{\text{W}}_{\lambda_{X}}{\text{X}}_{j}$ where ${\text{W}}_{\lambda_{X}}={\left({\text{X}}^{⊤}\text{X}+\lambda_{X}{\text{I}}_{p}\right)}^{-1}$ is the ridge-penalized weight matrix. The corresponding Gram matrix for this kernel is denoted by


(4) $$\begin{array}{l} {\text{H}}_{\lambda_{X}}=\text{X}{\left({\text{X}}^{⊤}\text{X}+\lambda_{X}{\text{I}}_{p}\right)}^{-1}{\text{X}}^{⊤}. \end{array}$$

We define K^**Y**^ and the associated Gram matrix **K**_λ_*Y*__ similarly using **Y**. The Mantel test statistic is equivalent to tr(**H**_λ_*X*__**K**_λ_*Y*__). Under the null hypothesis, there is no association between the similarities measured by K^**X**^ and K^**Y**^. In practice, the reference distribution can be obtained *via* a permutation procedure (Nichols and Holmes, 2002; Shaw and Proschan, 2013; Zhou et al., 2014). For instance, we can simultaneously permute the rows and columns of **Y**, while keeping **X** fixed. Equivalently, for a fixed matrix **H**_λ_*X*__, we can permute the observation labels for **K**_λ_*Y*__ and calculate the empirical null distribution.

### 2.2. Score Test in Linear Models

The general framework of Mantel Test presented in Section 2.1 encompasses several association tests (for examples, see Robert and Escoufier, 1976; Székely et al., 2007; Xu et al., 2017) and various kernel functions can be investigated to reflect model complexity and detect underlying linear or non-linear associations. Moreover, Pluta et al. (2021) developed a unified framework of linear models that links the Mantel test and Rao's score test (Rao, 1948) in a class of tests indexed by the ridge penalty. Following the discussion of Pluta et al. (2021), we consider the following linear models:

1. Fixed Effects Model: **Y** = **X*****β*** + **ε**, where **ε** ~ *N*(**0**, **I**_*n*_, **Σ**) or alternatively using the vectorized response variables, vec(**Y**) ~ *N*(vec(**X*****β***), **Σ** ⊗ **I**_*n*_) where vec(·) is the vectorization operator and ⊗ refers to the Kronecker product operator on two matrices.
2. Random Effects Model: **Y** = **Xb** + **ε** where **ε** ~ *N*(**0**, **I**_*n*_, **Σ**_*q*_) and **b** ~ *N*(**0**, **I**_*p*_, **Σ**_*b*_) or equivalently, $\text{vec}\left(\text{Y}\right)\sim N\left(0,\Sigma_{b}⊗\text{X}{\text{X}}^{⊤}+\Sigma ⊗{\text{I}}_{n}\right)$.

To describe the score statistic compactly, we consider the Singular Value Decomposition (SVD) of the matrix **X = UDV**^⊤^, where **U** and **V** are orthogonal, and **D** is a diagonal matrix with the (non-negative) singular values. To perform the global test *H*_0_:***β*** = **0** under the fixed effects model, the score test statistic is given by


(5) $$\begin{array}{l} S_{FE}≍{\text{Y}}^{⊤}\text{X}{\left({\text{X}}^{⊤}\text{X}\right)}^{-}\left({\text{X}}^{⊤}\text{Y}\right)=\text{tr}\left(\text{Z}{\text{Z}}^{⊤}\right)=\overset{r}{\underset{j=1}{\sum }}Z_{j}^{2}\overset{H_{0}}{\sim }\overset{r}{\underset{j=1}{\sum }}\chi_{1,j}^{2} \end{array}$$

where **Z** = **U**^⊤^**Y** and *r* = rank(**X**). The notation **A**^−^ denotes the Moore-Penrose pseudoinverse of the matrix **A**. It is well-known that the Moore-Penrose psedoinverse leads to the minimum norm solution to the least-squares problem. On the other hand, to test *H*_0_:**Σ**_*b*_ = **0** under certain conditions, the score test statistic for the random effects (variance components) model is


(6) $$\begin{array}{l} S_{RE}≍{\text{Y}}^{⊤}\text{X}\left({\text{X}}^{⊤}\text{Y}\right)=\text{tr}\left({\text{Z}}^{⊤}\text{D}{\text{D}}^{⊤}\text{Z}\right)=\overset{r}{\underset{j=1}{\sum }}d_{j}^{2}Z_{j}^{2}\overset{H_{0}}{\sim }\overset{r}{\underset{j=1}{\sum }}d_{j}^{2}\chi_{1,j}^{2}. \end{array}$$

Finally, the ridge regression score test statistic for testing *H*_0_:***β*** = **0** is


(7) $$\begin{array}{l} S_{RR}≍{\text{Y}}^{⊤}\text{X}{\left({\text{X}}^{⊤}\text{X}+\lambda_{X}{\text{I}}_{p}\right)}^{-1}\left({\text{X}}^{⊤}\text{Y}\right)\\ \;=\text{tr}\left({\text{Z}}^{⊤}\text{D}{\left({\text{D}}^{⊤}\text{D}+\lambda_{X}{\text{I}}_{p}\right)}^{-1}{\text{D}}^{⊤}\text{Z}\right). \end{array}$$

Hence,


(8) $$\begin{array}{l} \overset{r}{\underset{j=1}{\sum }}\frac{d_{j}^{2}}{d_{j}^{2}+\lambda_{X}}Z_{j}^{2}\overset{H_{0}}{\sim }\overset{r}{\underset{j=1}{\sum }}\frac{d_{j}^{2}}{d_{j}^{2}+\lambda_{X}}\chi_{1,j}^{2}. \end{array}$$

As summarized in Pluta et al. (2021), the score test statistics described in (5) – (7) can be formulated equivalently as tr(**H**_λ_*X*__**K**_λ_*Y*__) which is the expression for the Mantel test statistic described in Section 2.1. In particular, the fixed effects score test statistic is equivalent to $\text{tr}\left({\text{H}}_{0}\text{Y}{\text{Y}}^{⊤}\right)$ where ${\text{H}}_{0}=\text{X}{\left({\text{X}}^{⊤}\text{X}\right)}^{-}{\text{X}}^{⊤}$. Meanwhile, when $\Sigma =\sigma^{2}{\text{I}}_{q}$ and $\Sigma_{b}=\sigma_{b}^{2}{\text{I}}_{q}$, then the score statistic corresponding to the random effects model is proportional to tr(**H**_∞_**K**_∞_) where ${\text{H}}_{\infty }=\text{X}{\text{X}}^{⊤}$ and ${\text{K}}_{\infty }=\text{Y}{\text{Y}}^{⊤}$ (Pluta et al., 2021). Lastly, the ridge regression score test statistic can be written as $\text{tr}\left({\text{H}}_{\lambda_{X}}\text{Y}{\text{Y}}^{⊤}\right)$ using **H**_λ_*X*__ provided in (4). Furthermore, Pluta et al. (2021) highlights that the ridge regression score statistic is a compromise between the fixed effects and variance components tests. For small values of the ridge penalty λ_*X*_, the test statistic in (7) approaches the fixed effects score test statistic, and is identical at λ_*X*_ = 0. Also, Pluta et al. (2021) remarked that a large choice of λ_*X*_ yields a test close to the random effects score statistic, converging to identical tests as λ_*X*_ → ∞.

### 2.3. Examining the Choice of Ridge Penalty Parameter

Motivated by the framework introduced by Pluta et al. (2021), which categorizes the association test and score tests into a single class of tests characterized by the ridge penalty, we examine the choice of this parameter in the high-dimensional hypothesis testing set-up. In practice, the optimal choice of ridge penalty parameter is based on the observed data and proper data-dependent tuning is among the central tasks in statistical learning (Patil et al., 2021).

#### 2.3.1. Ridge Predictive Performance

The role of the ridge penalty in high-dimensional prediction and estimation has been an active area of research in the past several years. For both asymptotic and non-asymptotic settings, the predictive performance of ridge regression has been studied extensively (see Hsu et al., 2012; Cule and De Iorio, 2013; Karoui, 2013; Dobriban and Wager, 2018; Hastie et al., 2019; Wu and Xu, 2020; Richards et al., 2021 for examples). Furthermore, Kobak et al. (2020) demonstrated that an explicit positive ridge penalty can fail to provide any improvement over the minimum-norm least squares estimator using simulations and real-life high-dimensional data sets. In particular, they showed that the optimal value of ridge penalty in this situation could be negative when *n* ≪ *p*. Similar to these work, in this article, we focus on the role of ridge penalty in hypothesis testing for a univariate response, i.e., *q* = 1. The extension of to multivariate responses will be considered in future research. In Sections 2.1 and 2.2, λ_*X*_ corresponds to the tuning parameter in the Gram matrix of the ridge kernel associated with **X** which is not necessarily the same as λ_*Y*_, the tuning parameter in the ridge kernel corresponding to **Y**. However, under the univariate response **y** setting, we only have to specify the ridge penalty parameter λ_*X*_. For brevity, we will refer to λ_*X*_ as λ in the next sections.

#### 2.3.2. Ridge Cross-Validation

The performance of the fitted model is affected by the calibration of the regularization parameter. One of the most widely used methods for regularization tuning is cross-validation (for examples, see Allen, 1971; Stone, 1974; Delaney and Chatterjee, 1986; Arlot and Celisse, 2010). In ridge regression, two commonly used cross-validation procedures are generalized cross-validation (GCV) (Golub et al., 1979) and leave-one-out cross-validation (LOOCV), a variant of the *k*-fold cross-validation (Hastie et al., 2009). GCV, a rotation-invariant version of the predicted residual error sum of squares (PRESS), is a popular choice in practice because it does not require model refitting. Similarly, approximation methods to LOOCV (e.g., Kumar et al., 2013; Meijer and Goeman, 2013) to circumvent the problem of computational complexity brought by multiple model refitting.

The LOOCV estimate for a response vector **y** containing *n* observations is defined as


(9) $$\begin{array}{l} \text{loocv}\left(\lambda \right)=\frac{1}{n}\overset{n}{\underset{i=1}{\sum }}{\left(y_{i}-{\text{X}}_{i}^{⊤}{\hat{\beta }}_{-i,\lambda }\right)}^{2} \end{array}$$

where ${\hat{\beta }}_{-i,\lambda }$ is the ridge estimate when the *i*th observation is not included in the training set. As cited in Patil et al. (2021), an alternative formula for the LOOCV (Hastie et al., 2009) is given by


(10) $$\begin{array}{l} \text{loocv}\left(\lambda \right)=\frac{1}{n}\overset{n}{\underset{i=1}{\sum }}{\left(\frac{y_{i}-{\text{X}}_{i}^{⊤}{\hat{\beta }}_{\lambda }}{1-{\left[{\text{H}}_{\lambda }\right]}_{ii}}\right)}^{2} \end{array}$$

where [_**H**_λ_]*ii*_ corresponds to the *i*th diagonal entry of the matrix ${\text{H}}_{\lambda }=\text{X}{\left({\text{X}}^{⊤}\text{X}+\lambda {\text{I}}_{p}\right)}^{-}{\text{X}}^{⊤}$. Closely similar to (10), the GCV estimate formulation provided by Patil et al. (2021) is


(11) $$\begin{array}{l} \text{gcv}\left(\lambda \right)=\frac{1}{n}\overset{n}{\underset{i=1}{\sum }}{\left(\frac{y_{i}-{\text{X}}_{i}^{⊤}{\hat{\beta }}_{\lambda }}{1-\text{tr}\left({\text{H}}_{\lambda }\right)/n}\right)}^{2} \end{array}$$

where the average of the trace elements is used instead of the *i*th diagonal entry. When λ = 0 and rank(**X**) = *n*, the diagonal elements of **H**_0_ is equal to 1 and tr(**H**_0_) reduces to *n*. In this case, the ridge regression is an interpolator of $\text{X}{\hat{\beta }}_{\lambda }=\text{y}$ (Patil et al., 2021). Since both numerator and denominator of the expressions in (10) and (11) are 0, Hastie et al. (2019) defined the LOOCV and GCV estimates based on the limits λ → 0, respectively.

Moreover, the asymptotic optimality of LOOCV and GCV tuning for ridge regression in high-dimensional setting is presented by Hastie et al. (2019). Patil et al. (2021) generalized the scope discussed in Hastie et al. (2019) by showing that the GCV converges almost surely to the expected out-of-sample prediction error, uniformly over a set of candidate ridge regularization parameters. The discussion provided by Patil et al. (2021) is aligned with Kobak et al. (2020) wherein the optimal ridge penalty parameter can be positive, negative, or zero.

### 2.4. Rationale and Illustration of Contributions of Our Work

The Adaptive Mantel test (AdaMant) coined by Pluta et al. (2021) is an extension of the classical Mantel Test by incorporating the ridge penalty parameter to association testing. The adaptive procedure involves the calculation of similarity matrices ${\text{H}}_{m}=K_{m}^{\text{X}}\left(\text{X}\right)$ and ${\text{K}}_{m}=K_{m}^{\text{Y}}\left(\text{Y}\right)$ for every pair of input metrics or kernels (K^**X**^, K^**Y**^), *m* = 1, 2, …, *M*. Under the null hypothesis of no association between the similarities measured by K^**X**^ and K^**Y**^, Pluta et al. (2021) proposed a permutation procedure where they generate *B* permutations of the observation labels for **H**_*m*_ for a fixed matrix **K**_*m*_. The *p*-value $P_{m}^{\left(b\right)}$ is computed as a function of the test statistic tr$\left({\text{H}}_{m}^{\left(b\right)}{\text{K}}_{m}\right)$ for each *m* = 1, 2, …, *M* and permutations *b* = 1, 2, …, *B*. Finally, Pluta et al. (2021) defined the AdaMant test statistic as $P^{\left(0\right)}=\underset{m\in \left\{1,2,\ldots ,M\right\}}{\min}P_{m}^{\left(0\right)}$ where *b* = 0 refers to the original data set. Using the permutation procedure to obtain the empirical null distribution of *P*^(0)^, the corresponding AdaMant *p*-value is the proportion of *P*^(*b*)^ less than or equal to *P*^(0)^, that is,


(12) $$\begin{array}{l} P_{\text{AdaMant}}=\frac{1}{B+1}\overset{B}{\underset{b=0}{\sum }}\text{I}\left(P^{\left(b\right)}\leq P^{\left(0\right)}\right) \end{array}$$

where $P^{\left(b\right)}=\underset{m\in \left\{1,2,\ldots ,M\right\}}{\min}P_{m}^{\left(b\right)}$.

However, the main limitation in the ridge-penalized AdaMant procedure by Pluta et al. (2021) is the optimal selection of the ridge penalty parameter. When kernels of the form $\text{X}{\left({\text{X}}^{⊤}\text{X}+\lambda {\text{I}}_{p}\right)}^{-1}{\text{X}}^{⊤}$ are considered in AdaMant, λ is chosen to be proportional to the signal-to-noise ratio re-expressed as a function of genetic heritability *h*^2^ and number of explanatory variables *p* (Pluta et al., 2021). This implies that the value of the chosen ridge penalty is restricted to be non-negative. In their examples, the ridge penalty is chosen from a set with only a few values such as λ ∈ {100, 1, 000, 2, 500, 5, 000, 7, 500, 25, 000, ∞}. With a limited number of ridge penalty parameters to choose from, the λ which yields the highest empirical power may not be captured by the initial study of Pluta et al. (2021).

**Figure 1:**
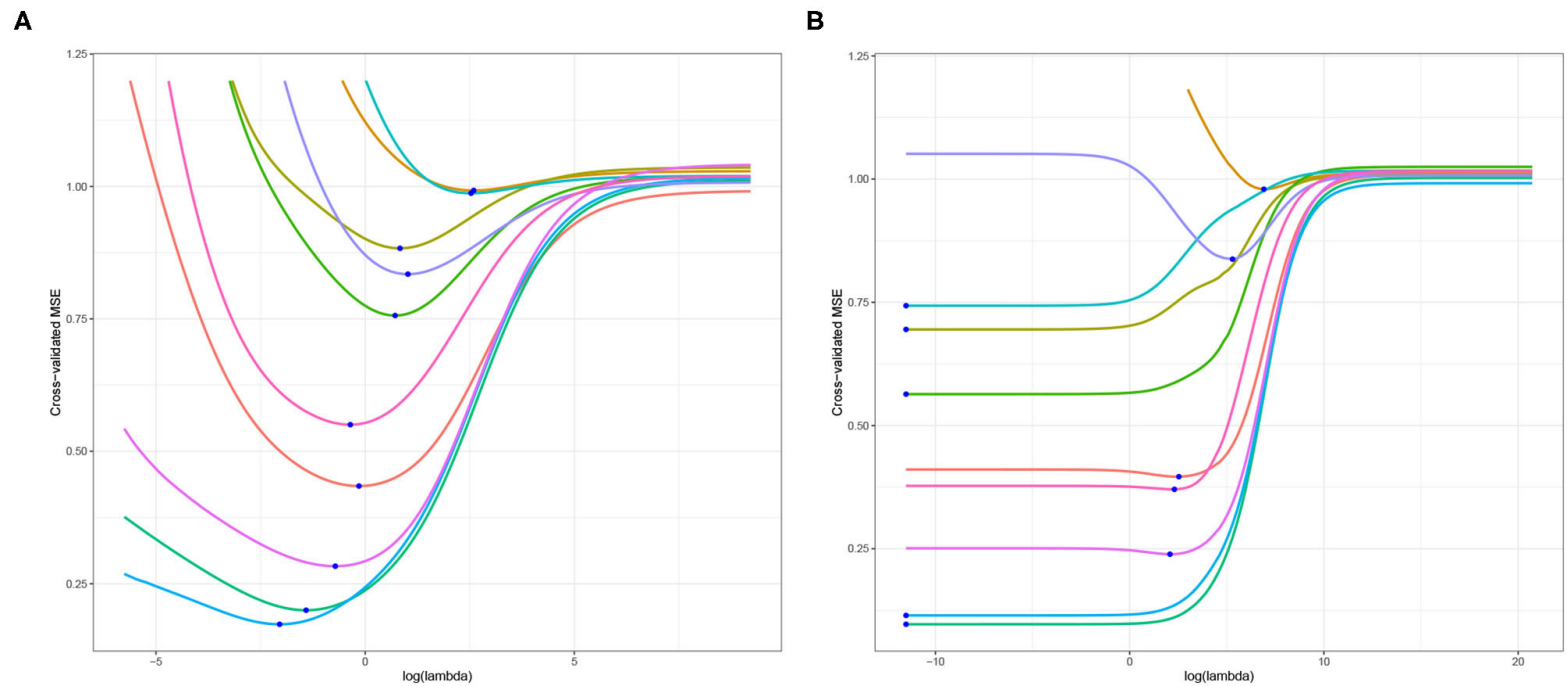
Cross-validated MSE of ridge regression using **(A)** *n* = 64 and *p* = 50 randomly selected explanatory variables; **(B)** *n* = 64 and *p* = 3, 116, all explanatory variables. The blue dot corresponds to the minimum cross-validated MSE for each dependent variable.

As highlighted in Section 1, the primary objective of this article is to examine the optimal choice of ridge penalty in the high-dimensional hypothesis testing scenario. To illustrate the utility of addressing this goal and our subsequent contributions, we study liver.toxicity data set in Bushel et al. (2007). This data contains microarray expression levels of *p* = 3, 116 genes and 10 clinical chemistry measurements in liver tissue of *n* = 64 rats. First, we replicate the results presented in Kobak et al. (2020) using 10-fold cross-validation for varying ridge penalty parameter λ using one dependent variable at a time. The cross-validated MSE plotted for each dependent variable is displayed in Figure 1. In Figure 1A where *n* > *p*, Kobak et al. (2020) showed that this result is in agreement with the seminal article by Hoerl and Kennard (1970) wherein the optimal penalty is always larger than zero under the low-dimensional setting. However, in Figure 1B, five out of ten dependent variables yielded a minimum cross-validated MSE corresponding to the smallest value of λ considered when *n* ≪ *p* (Kobak et al., 2020).

Motivated by the aforementioned results, we investigated the empirical power and average of the -log_10_ *p*-values of the Adaptive Mantel test for several values of λ. We employ the liver toxicity data as our motivating example because it has been widely used recently to better understand overfitting. It was found that the clinical variables may not facilitate in the detection of paracetamol toxicity in the liver, but gene expression could be an alternative solution (Heinloth et al., 2004; Bushel et al., 2007). In this illustration, we compute the empirical power for a fixed λ, using one dependent variable at a time. For each replication, we add a vector of random noise to the vector of response, that is, **y**_*s*_ = **y** + **ε**_*s*_ for *s* = 1, 2, …, *S*. Under the null hypothesis when ***β*** = 0, the linear model **y** = **X*****β*** + **ε** reduces to **y** = **ε**. Hence, we can view the recursive expression as **y**_*s*_ = (**X*****β*** + ***ε***) + ***ε***_*s*_ ≠ ***ε*** in favor of the case that the alternative hypothesis is true. For *s* = 1, 2, …, *S*, we compute the AdaMant *p*-value at each λ using **y**_*s*_ and the entire matrix of gene expression **X** as inputs to the ridge kernel described in (4). After repeating this procedure for a total of *S* replications, the empirical power is computed as the proportion of replications where the Adamant *p*-value is less than the nominal level of significance α.


(13) $$\begin{array}{l} {\text{Power}}_{\lambda }=\frac{1}{S}\overset{S}{\underset{s=1}{\sum }}\text{I}\left(P_{\text{AdaMant},\lambda ,s}\leq \alpha \right) \end{array}$$

**Figure 2:**
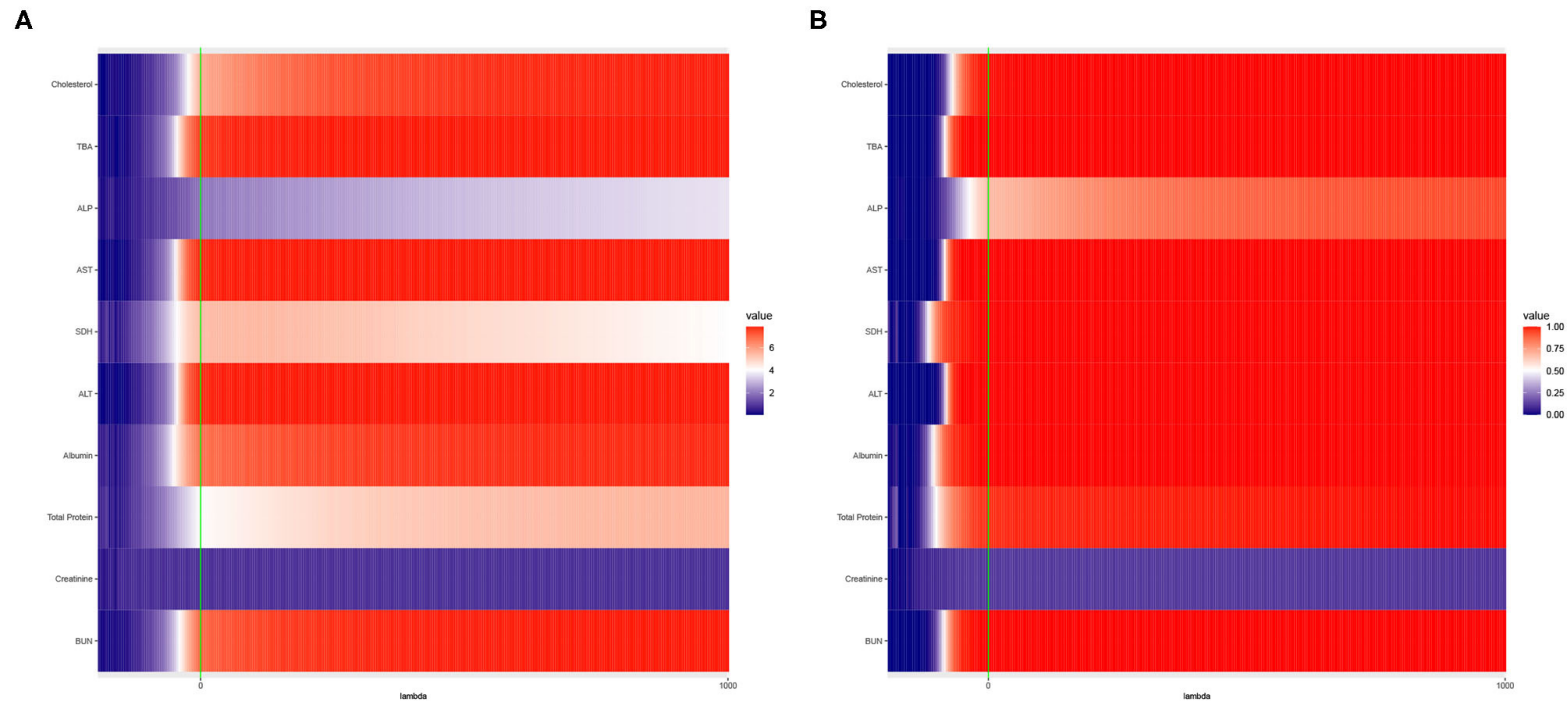
Heat maps of **(A)** Average of −log_10_ *p*-values and **(B)** Empirical Power of the Adaptive Mantel test using the liver toxicity data with *n* = 64 observations, *p* = 3, 116 genetic features. The green vertical line corresponds to λ = 0.

The results are presented in Figure 2.

To circumvent the limitations of the range of ridge penalty parameter considered in Pluta et al. (2021), we allowed the interval of λ to include negative, zero and positive values. According to Figure 2A, even though there is a more distinct gradient in the values of the average of the −log_10_ *p*-values compared to the empirical power in Figure 2B, eight out of ten dependent variables displayed more or less similar patterns in terms of empirical power. Also, based on Figure 2B, some λ < 0 lead to an empirical power approaching 1 when *n* ≪ *p*. This result is in alignment with the main result reported by Kobak et al. (2020) where the optimal ridge penalty for real-world high-dimensional data can be negative due to implicit ridge regularization. This phenomenon prompted us to further investigate real-valued ridge penalty parameters using imaging genetics data where the signals are weaker and sparsity is much more evident.

## 3. Methodology

In this section, we discuss the proposed methodology to incorporate the optimal selection of the ridge penalty parameter *via* cross-validation in the Adaptive Mantel test. The interval from which the optimal ridge penalty parameter will be selected from is discussed in Section 3.1 while the proposed methods are discussed in 3.2. The two algorithms to be compared are discussed thoroughly in Sections 3.2.1 and 3.2.3. Meanwhile, the features of the score test statistic are discussed in Section 3.2.2.

### 3.1. Range of Ridge Penalty Parameter

Before delving into the optimal choice of ridge penalty parameter to be used in hypothesis testing, it is crucial to specify the domain of sensible values first. Formally, we describe how to choose the interval I = (λ_min_, ∞) in this section. Following the third assumption in the main results of Patil et al. (2021), the minimum eigenvalue is bounded below by a constant ℓ_min_ > 0 where ℓ_min_ is independent of *p*. Patil et al. (2021) proposed that the smallest possible value of the regularization parameter wherein the GCV converges almost surely to LOOCV is given by $\lambda_{\min}=-{\left(\sqrt{p/n}-1\right)}^{2}\ell_{\min}$. However, Patil et al. (2021) did not clearly specify the value of ℓ_min_ apart from the constraint that it is positive.

In our proposed method, we extend the work of Patil et al. (2021) by relaxing the assumption on the minimum eigenvalue and allowing a fraction of eigenvalues to accumulate near zero. Specifically, we define a threshold on the small but non-zero singular values of *X*. Let *d*_1_ ≥ *d*_2_… ≥ *d*_*r*_ denote the non-negative singular values. To identify λ_min_, we first compute the adjusted singular values as follows


$${\tilde{d}}_{j}=\{\begin{array}{ll} d_{j} & d_{j}\geq \tau \\ 0 & d_{j}<\tau \end{array}$$

where τ is some threshold based on the quantiles. A generic choice for τ is the median of the singular values (Bühlmann and Ćevid, 2020). Next, we define $\ell_{\min}=\min\left\{\tilde{d_{j}}:\tilde{d_{j}}>0\right\}$. The value of ℓ_min_ coincides with τ if the value of the quantile is equal to one of the singular values. Otherwise, ℓ_min_ is the singular value greater than and closest to the quantile. We then compute $\lambda_{\min}=-{\left(\sqrt{p/n}-1\right)}^{2}\ell_{\min}$ where the range of λ allows for negative values including zero, when *p* ≠ *n*. We will investigate several values of τ and how it affects both Type I error rate and empirical power in the numerical studies section.

### 3.2. Proposed Methods

We extend the Adaptive Mantel Test (AdaMant) by Pluta et al. (2021) to include the optimal selection of the ridge penalty parameter *via* cross-validation in this section. For simplicity, we will refer to this proposed procedure as AdaMantCV. By using a permutation procedure on the set of test statistics, this procedure can simultaneously test across a set of ridge penalty parameters without increasing the Type I error rate (Pluta et al., 2021). Prior to the analysis, centering and scaling the explanatory and response variables is necessary because they lead to potential computational efficiency and stability and conceptual simplicity. More importantly, performing this ensures that penalty term will have an similar effect on all coefficient estimates.

#### 3.2.1. Adaptive Mantel Test With Cross-Validation

Under the null hypothesis of no association between the variation in a set of candidate SNPs and the variation in brain imaging data, say EEG coherence, we will implement a permutation procedure where *B* permutations are generated from rows of **y** for a fixed matrix **X**. Since the matrix **X** does not vary across the permutations generated, we only need to perform SVD once to obtain the vector of singular values for the calculation of the weights and specification of the interval of ridge penalty parameters.

We start by specifying the number of possible values of the ridge penalty parameter λ ∈ I, denoted by *M* and the threshold τ. Using these inputs coupled with the singular values, we implement the proposed method in Section 3.1 to identify the interval I = (λ_min_, ∞). Unlike Pluta et al. (2021), the proposed algorithm only require the kernel $K_{\lambda }^{\text{X}}$ as input because we only consider the univariate response **y** in this study. For $K_{\lambda }^{\text{X}}$, we include a single family of kernels with varying ridge penalty parameters. For a given cross-validation measure, say GCV, we compute the optimal value of the ridge penalty parameter using **y**^(*b*)^ and **X** as


(14) $$\begin{array}{l} {\hat{\lambda }}^{\left(b\right)}=\underset{\lambda \in I}{\text{argmin}}\text{gcv}\left(\lambda \right),b=0,1,2,\ldots ,B \end{array}$$

where *b* = 0 is associated to the original data set. For each *b* = 0, 1, 2, …, *B*, we also compute the ridge regression score test statistic $T^{\left(b\right)}\left({\hat{\lambda }}^{\left(b\right)}\right)$ given by tr$\left({\text{H}}_{{\hat{\lambda }}^{\left(b\right)}}{\text{K}}^{\left(b\right)}\right)$ where ${\text{H}}_{{\hat{\lambda }}^{\left(b\right)}}=\text{X}{\left({\text{X}}^{⊤}\text{X}+{\hat{\lambda }}^{\left(b\right)}{\text{I}}_{p}\right)}^{-1}{\text{X}}^{⊤}$.

To account for varying magnitudes of the quantity $T^{\left(b\right)}\left({\hat{\lambda }}^{\left(b\right)}\right)$ for different values of ${\hat{\lambda }}^{\left(b\right)}$, our proposed AdaMantCV test statistic *P*^(0)^ is transformed to take on values between 0 and 1, inclusive. This test statistic is computed as follows


(15) $$\begin{array}{l} P^{\left(0\right)}=\frac{1}{B+1}\overset{B}{\underset{b=0}{\sum }}\text{I}\left(T^{\left(0\right)}\left({\hat{\lambda }}^{\left(0\right)}\right)\leq T^{\left(b\right)}\left({\hat{\lambda }}^{\left(0\right)}\right)\right). \end{array}$$

This indicates that for a fixed value of ${\hat{\lambda }}^{\left(0\right)}$, we want to compare the magnitude of the observed test statistic from the original data vs. the computed test statistics using the permuted data. Finally, the analogous AdaMantCV *p*-value is the proportion of *P*^(*b*)^ no greater than *P*^(0)^, that is,


(16) $$\begin{array}{l} P_{\text{CV}}=\frac{1}{B+1}\overset{B}{\underset{b=0}{\sum }}\text{I}\left(P^{\left(b\right)}\leq P^{\left(0\right)}\right),\text{where}\\ P^{\left(b\right)}=\frac{1}{B+1}\overset{B}{\underset{c=0}{\sum }}\text{I}\left(T^{\left(b\right)}\left({\hat{\lambda }}^{\left(b\right)}\right)\leq T^{\left(c\right)}\left({\hat{\lambda }}^{\left(b\right)}\right)\right). \end{array}$$

The general pseudocode of the AdaMantCV algorithm is presented below. The limitation of the straightforward application of this procedure is that it is computationally expensive when *n* ≪ *p* (Pluta et al., 2021). To deal with this, following Pluta et al. (2021), we utilized the identity presented in Henderson and Searle (1981) and Kobak et al. (2020).


(17) $$\begin{array}{l} {\left({\text{X}}^{⊤}\text{X}+\lambda {\text{I}}_{p}\right)}^{-1}{\text{X}}^{⊤}={\text{X}}^{⊤}{\left(\text{X}{\text{X}}^{⊤}+\lambda {\text{I}}_{n}\right)}^{-1} \end{array}$$

wherein the dimension of the matrix to be inverted is *n* × *n* instead of *p* × *p*. Additionally, Pluta et al. (2021) has shown that the Mantel test statistic has a complexity of *O*(*n*^2^) and coupled with *B* permutations, the total computational complexity is *O*(*n*^2^*p* + *n*^2^*B*), which is less than the required computational complexity using SVD.

**Table:** 

1: **procedure** Adaptive Mantel test with CV:($\text{X},\text{y},K_{\lambda }^{\text{X}},\text{CV}\left(\lambda \right),\tau ,M,B$)
2: Specify the interval I as a function of (**X**, τ, *M*)
3: Calculate ${\hat{\lambda }}^{\left(0\right)}:=\underset{\lambda \in I}{\text{argmin}}\text{CV}\left(\lambda \right)$
4: Calculate $T^{\left(0\right)}\left({\hat{\lambda }}^{\left(0\right)}\right)\leftarrow \text{tr}\left[{\text{H}}_{{\hat{\lambda }}^{\left(0\right)}}\text{K}\right]$ where ${\text{H}}_{{\hat{\lambda }}^{\left(0\right)}}=\text{X}{\left({\text{X}}^{⊤}\text{X}+{\hat{\lambda }}^{\left(0\right)}{\text{I}}_{p}\right)}^{-1}{\text{X}}^{⊤}$ and denote **K** = **yy**^⊤^
5: Generate *B* permutations of **y**, labeled **y**^(*b*)^, and denote **K**^(*b*)^ = **y**^(*b*)^(**y**^(*b*)^)⊤∀*b* = 1, …, *B*
6: Calculate ${\hat{\lambda }}^{\left(b\right)}:=\underset{\lambda \in I}{\text{argmin}}\text{CV}\left(\lambda \right)$
7: $T^{\left(b\right)}\left({\hat{\lambda }}^{\left(b\right)}\right)\leftarrow \text{tr}\left[{\text{H}}_{{\hat{\lambda }}^{\left(b\right)}}{\text{K}}^{\left(b\right)}\right]\;\forall b=1,\ldots ,B$ where ${\text{H}}_{{\hat{\lambda }}^{\left(b\right)}}=\text{X}{\left({\text{X}}^{⊤}\text{X}+{\hat{\lambda }}^{\left(b\right)}{\text{I}}_{p}\right)}^{-1}{\text{X}}^{⊤}$
8: $T^{\left(c\right)}\left({\hat{\lambda }}^{\left(b\right)}\right)\leftarrow \text{tr}\left[{\text{H}}_{{\hat{\lambda }}^{\left(b\right)}}{\text{K}}^{\left(c\right)}\right]\;\forall c=0,1,\ldots ,B$
9: $P^{\left(b\right)}\leftarrow \frac{1}{B+1}\overset{B}{\underset{c=0}{\sum }}\text{I}\left(T^{\left(b\right)}\left({\hat{\lambda }}^{\left(b\right)}\right)\leq T^{\left(c\right)}\left({\hat{\lambda }}^{\left(b\right)}\right)\right)\forall b,c=0,1,\ldots ,B$
10: $P_{\text{CV}}\leftarrow \frac{1}{B+1}\overset{B}{\underset{b=0}{\sum }}\text{I}\left(P^{\left(b\right)}\leq P^{\left(0\right)}\right)$
11: **end procedure**

#### 3.2.2. Features of the Score Test Statistic in AdaMantCV

The ridge regression score test statistic $T^{\left(b\right)}\left({\hat{\lambda }}^{\left(b\right)}\right)$ = tr$\left({\text{H}}_{{\hat{\lambda }}^{\left(b\right)}}{\text{K}}^{\left(b\right)}\right)$ can be expressed equivalently as


(18) $$\begin{array}{l} T^{\left(b\right)}\left({\hat{\lambda }}^{\left(b\right)}\right)=\overset{r}{\underset{j=1}{\sum }}\frac{d_{j}^{2}}{d_{j}^{2}+{\hat{\lambda }}^{\left(b\right)}}{\left[Z_{j}^{\left(b\right)}\right]}^{2}=\overset{r}{\underset{j=1}{\sum }}w_{j}\left({\hat{\lambda }}^{\left(b\right)}\right){\left[Z_{j}^{\left(b\right)}\right]}^{2} \end{array}$$

where $Z_{j}^{\left(b\right)}={\text{U}}_{j}^{⊤}{\text{y}}^{\left(b\right)}$, as described in (8). From the specification of the test statistic in (18), the weights are non-negative if the set of possible values of λ is restricted to the positive range only. More importantly, $0<w_{j}\left({\hat{\lambda }}^{\left(b\right)}\right)<1$ for non-zero singular values *d*_*j*_ which indicates that the test statistic components shrink toward zero if we choose a large positive value of the ridge penalty parameter. This means that for a fixed ${\left[Z_{j}^{\left(b\right)}\right]}^{2}>0$, the weight $w_{j}\left({\hat{\lambda }}^{\left(b\right)}\right)$ corresponding to the *j*th component of the score test statistic $T^{\left(b\right)}\left({\hat{\lambda }}^{\left(b\right)}\right)$ has the following features:

(i) $w_{j}\left({\hat{\lambda }}^{\left(b\right)}\right)\to 0$ if ${\hat{\lambda }}^{\left(b\right)}\to \infty$. However, as argued in Pluta et al. (2021), the standardized version essentially indicates that $w_{j}\left(\infty \right)\propto d_{j}^{2}$;
(ii) $w_{j}\left({\hat{\lambda }}^{\left(b\right)}\right)=1$, i.e., equal weights, if ${\hat{\lambda }}^{\left(b\right)}=0$; and
(iii) $w_{j}\left({\hat{\lambda }}^{\left(b\right)}\right)>1$ and, smaller but a positive $d_{j}^{2}$ weight leads to a larger weight in a relative sense if ${\hat{\lambda }}^{\left(b\right)}<0$ provided $d_{j}^{2}+{\hat{\lambda }}^{\left(b\right)}>0$.

This suggests that in the context of Adaptive Mantel Test in high-dimensional setting, a negative choice of penalty parameter has potential in achieving superior empirical power when $Z_{j}^{2}$ tends to associate with directions of low-variance where variance is measured by the eigenvalues of variance-covariance matrix of the covariates.

Another crucial consideration for the score test statistic in (18) is that the weights should be non-negative for all *j* = 1, 2, …*r* = min(*n, p*) because the statistic is asymptotically distributed as a mixture of chi-squared random variables. If ${\hat{\lambda }}^{\left(b\right)}\geq 0$, then there is no constraint about the form of the test statistic. However, if ${\hat{\lambda }}^{\left(b\right)}<0$, then we should ensure that the all the weights remain positive to satisfy the asymptotic distributional assumptions. A simple and straightforward strategy to handle negative weights is to use the adjusted weights defined as ${\tilde{w}}_{j}=\max\left(w_{j},0\right)$. In addition, if ${\hat{\lambda }}^{\left(b\right)}=0$ and *d*_*j*_ = 0, we will utilize the formula for the fixed effects score test statistic mentioned in (5) to avoid the case where both the numerator and denominator of (18) is zero.

We can also show mathematically that for any *j* = 1, 2, …, *r*, the optimal value of the ridge penalty parameter must satisfy the following condition


$$\begin{array}{l} {\hat{\lambda }}^{\left(b\right)}>\underset{1\leq j\leq r}{\max}-d_{j}^{2} \end{array}$$

to ensure that the weights are non-negative. This shows that a potential choice for $\lambda_{\min}^{⋆}=-d_{\left(1\right)}^{2}+\epsilon$, where $d_{\left(1\right)}^{2}$ is the smallest eigenvalue and ϵ > 0. The value of ϵ is chosen to be the machine tolerance error in a statistical software. Formally, ϵ is the smallest positive floating-point number such that 1 + ϵ ≠ 1. However, in the high-dimensional setting, the smallest eigenvalue is very close to zero and could even be lower than the machine tolerance ϵ. This may lead to the case that $\lambda_{\min}^{⋆}>0$, i.e., the interval of ridge penalty parameters include positive values only. To address the objective of investigating the role of real-valued ridge penalty parameters in high-dimensional hypothesis testing, our focus in this study is on intervals I where the lower bound is negative.

#### 3.2.3. Adaptive Mantel Test With Gamma Approximation and Cross-Validation

Alternatively, we can use the *B* permutations to estimate the parameters of the asymptotic null distribution. As mentioned previously, test statistic described in (8) is asymptotically distributed as a mixture of chi-squared random variables. To characterize this null distribution, we will use the Gamma distribution instead because it captures a general family of distributions where the chi-squared distribution is a special case. For a fixed λ ∈ I, suppose *T*^(1)^(λ), *T*^(2)^(λ), …, *T*^(*B*)^(λ) is a random sample from Gamma distribution under the null hypothesis. We compute the parameter estimates $\hat{\alpha }\left(\lambda \right)$ and $\hat{\beta }\left(\lambda \right)$ using Method of Moments for each λ ∈ I.

Following the classical definition of *p*-value $P^{\left(b\right)}\left({\hat{\lambda }}^{\left(b\right)}\right)$, we compute for the probability of $T^{\left(b\right)}\left({\hat{\lambda }}^{\left(b\right)}\right)$ is at least as large as the observed value $t^{\left(b\right)}\left({\hat{\lambda }}^{\left(b\right)}\right)$ when the null hypothesis is true, that is,


(19) $$\begin{array}{l} P^{\left(b\right)}\left({\hat{\lambda }}^{\left(b\right)}\right)={\mathbb{P}}_{H_{0}}\left[T^{\left(b\right)}\left({\hat{\lambda }}^{\left(b\right)}\right)\geq t^{\left(b\right)}\left({\hat{\lambda }}^{\left(b\right)}\right)\right]. \end{array}$$

The null distribution used in (19) is Gamma, with plug-in estimators for the shape and rate parameters denoted by $\hat{\alpha }\left({\hat{\lambda }}^{\left(b\right)}\right)$ and $\hat{\beta }\left({\hat{\lambda }}^{\left(b\right)}\right)$, respectively. The test statistic for this procedure is given by *P*^(0)^ which is the *p*-value in (19) associated with the optimal ridge penalty parameter ${\hat{\lambda }}^{\left(0\right)}$ for the original data. Similar to AdaMantCV, the *p*-value of this test can be computed using the proportion of $P^{\left(b\right)}=P^{\left(b\right)}\left({\hat{\lambda }}^{\left(b\right)}\right)$ less than or equal to *P*^(0)^ for all *b* = 0, 1, …, *B*. The general pseudocode of the Adaptive Mantel Test with Gamma Approximation and Cross-Validation (AdaMantGACV) algorithm is presented below.

**Table:** 

1: **procedure** Adaptive Mantel Test with Gamma Approx and CV: ($\text{X},\text{y},K_{\lambda }^{\text{X}},\text{CV}\left(\lambda \right),\tau ,M,B$)
2: Specify the interval I as a function of (**X**, τ, *M*)
3: Generate *B* permutations of **y**, labeled **y**^(*b*)^, ∀*b* = 0, 1, …, *B*. Denote **K**^(*b*)^ = *y*^(*b*)^(*y*^*b*^)⊤.
4: Calculate ${\hat{\lambda }}^{\left(b\right)}:=\underset{\lambda \in I}{\text{argmin}}\text{CV}\left(\lambda \right)\forall b=0,1,\ldots ,B$
5: $T^{\left(b\right)}\left({\hat{\lambda }}^{\left(b\right)}\right)\leftarrow \text{tr}\left[{\text{H}}_{{\hat{\lambda }}^{\left(b\right)}}{\text{K}}^{\left(b\right)}\right]\;\forall b=0,1,\ldots ,B$
6: $P^{\left(b\right)}\left({\hat{\lambda }}^{\left(b\right)}\right)\leftarrow {\mathbb{P}}_{H_{0}}\left[T^{\left(b\right)}\left({\hat{\lambda }}^{\left(b\right)}\right)\geq t^{\left(b\right)}\left({\hat{\lambda }}^{\left(b\right)}\right)\right]\;\forall b=0,1,\ldots ,B$
7: $P^{\left(b\right)}\leftarrow P^{\left(b\right)}\left({\hat{\lambda }}^{\left(b\right)}\right)\forall b=0,1,\ldots ,B$
8: $P_{\text{GACV}}\leftarrow \frac{1}{B+1}\overset{B}{\underset{b=0}{\sum }}\text{I}\left(P^{\left(b\right)}\leq P^{\left(0\right)}\right)$
9: **end procedure**

## 4. Results

### 4.1. Simulation Studies

To gain insights regarding the performance of the proposed procedures in terms of the correct and incorrect rejections, we perform some simulation studies. The comparison is based on four simulation settings:

(i) Number of explanatory variables *p*
(ii) Covariance structure of the simulated design matrix **X**
(iii) True linear model specification where **y** is generated from, and
(iv) Quantile of the singular values to be used as threshold τ.

To mimic the characteristics of the real data set, we consider *n* = 350 subjects and number of explanatory variables *p* as either 500 or 1000. The design matrix **X** is generated from multivariate normal distribution *N*(**0**_*p*_, **Σ**_*X*_) where the covariance structure is either heteroskedastic or compound symmetric. For the heteroskedastic covariance structure is **Σ**_*X*_ = **G**_*p*_ where the *j*th diagonal entry is *g*_*j*_ = log(*j* + 1), *j* = 1, 2, …, *p*. Likewise, the compound symmetric structure **Σ**_*X*_ is characterized by ρ_*X*_ = 0.025.

As discussed in Section 2.2, we are also interested in comparing the empirical power of the proposed methods when the vector of responses are generated using either the fixed effects or random effects model assumption. For the fixed effects model under the alternative hypothesis, the coefficients are ***β*** = ξ**1**_*p*_ while $\text{b}\sim N\left(0,\sigma_{b}^{2}\right)$ for the random effects model where ξ = 3 and σ_*b*_ = 0.50. Finally, we also want to explore whether the threshold τ have an impact on both the empirical power and the Type I error. In particular, we compare the scenarios wherein τ = 0, that is, no thresholding is implemented vs. the setting wherein we use the value of the first quartile (*Q*_1_) as well as the median or second quartile (*Q*_2_) of the singular values as the threshold.

There are four methods to be compared. For the Adaptive Mantel Test with Cross-Validation, we will implement it using GCV or LOOCV to select the optimal ridge penalty parameter. Similarly, we will implement the Adaptive Mantel Test with Gamma Approximation and Cross-Validation using these two cross-validation techniques, denoted as GAGCV and GALOOCV, respectively. For each simulation setting, 500 replications were run to estimate both the Type I error and the empirical power. A total of *B* = 1000 permutations and *M* = 250 values of λ were considered for each replication. Relative to the simulation studies in Pluta et al. (2021), we consider a more exhaustive range and selection of the ridge penalty parameter.

When the null hypothesis is true, the Type I error rate is computed empirically as


(20) $$\begin{array}{l} \text{Type I error}=\frac{1}{S}\overset{S}{\underset{s=1}{\sum }}\text{I}\left(P_{\text{CV},s}\leq \alpha \right) \end{array}$$

where *P*_CV, *s*_ represents the *p*-value of the Adaptive Mantel test with either GCV, LOOCV, GAGCV, or GALOOCV for the *s*th replication. On the other hand, when the null hypothesis is not true, the empirical power is computed as the proportion of correct rejections. Throughout the simulations, we consider the level of significance α = 0.05.

Results from Table 1 show the numerical comparison of the Type I error of the Adaptive Mantel test vs. the AdaMant with Gamma Approximation test implementing the optimal ridge penalty selection *via* generalized or leave-one-out cross-validation. Given that both algorithms are permutation-based, they naturally control the Type I error rate at any specified nominal level of significance α. Even though the *p*-values *P*_GCV_ and *P*_LOOCV_ obtained using AdaMantCV vary, the resulting Type I error rates using either GCV or LOOCV are more or less similar, and the proportion of incorrect rejections is controlled. A similar pattern is observed for the *p*-values computed from the AdaMant with Gamma Approximation algorithm for both cross-validation methods. Overall, the results presented in Table 1 verify that the proportion of incorrect rejections was controlled appropriately using the proposed methods.

**Table 1:** Numerical comparison of the Type I error of Adaptive Mantel test (i) with cross-validation vs. (ii) with Gamma approximation and cross-validation using the simulated data with *n* = 350, error standard deviation σ = 0.50 and ***β*** = **0**.

			**Adaptive Mantel Test**			
			**With CV**		**With GA and CV**	
**Covariance structure**	**p**	**τ**	**GCV**	**LOOCV**	**GCV**	**LOOCV**
Compound symmetric	500	None	0.042	0.042	0.036	0.036
		*Q*_1_	0.030	0.028	0.032	0.028
		*Q*_2_	0.048	0.050	0.052	0.052
	1,000	None	0.048	0.048	0.038	0.038
		*Q*_1_	0.044	0.044	0.042	0.042
		*Q*_2_	0.036	0.036	0.036	0.036
Heteroskedastic	500	None	0.038	0.038	0.038	0.038
		*Q*_1_	0.026	0.026	0.028	0.028
		*Q*_2_	0.046	0.046	0.050	0.050
	1,000	None	0.050	0.050	0.040	0.040
		*Q*_1_	0.046	0.046	0.046	0.046
		*Q*_2_	0.040	0.040	0.038	0.038

Tables 2, 3 provide a comparison of empirical power when the response data are generated from the fixed effects model and random effects model, respectively. Results revealed that there is an improvement in the empirical power when Gamma approximation is added to the Adaptive Mantel test with cross-validation. Given that both class of methods, AdaMantCV and AdaMantGACV, control the proportion of false rejections, the higher empirical power exhibited by AdaMantGACV indicate that it is the better method. It is also apparent from both tables that either AdaMantCV or AdaMantGACV leads to a superior empirical power, i.e., approaching 1, when *p* = 1,000. In contrast, both AdaMantCV and AdaMantGACV result to a lower empirical power when *p* = 500, regardless of the covariance structure used for generating the design matrix **X**. This result is supported by Hastie et al. (2019) and Patil et al. (2021) where statistical inference is the most challenging when *p*/*n* ≈ 1, compared to *p* ≪ *n* or *n* ≪ *p*.

**Table 2:** Numerical comparison of the Empirical Power of Adaptive Mantel test (i) with cross-validation vs. (ii) with Gamma approximation and cross-validation using the simulated data with *n* = 350, error standard deviation σ = 1 and ***β*** = ξ**1**_*p*_, ξ = 3.

			**Adaptive Mantel Test**			
			**With CV**		**With GA and CV**	
**Covariance structure**	**p**	**τ**	**GCV**	**LOOCV**	**GCV**	**LOOCV**
Compound symmetric	500	None	0.062	0.062	0.104	0.104
		*Q*_1_	0.342	0.290	0.378	0.326
		*Q*_2_	0.348	0.288	0.382	0.324
	1,000	None	0.998	0.998	0.998	0.998
		*Q*_1_	1.000	1.000	1.000	1.000
		*Q*_2_	1.000	1.000	1.000	1.000
Heteroskedastic	500	None	0.064	0.064	0.178	0.178
		*Q*_1_	0.074	0.072	0.186	0.184
		*Q*_2_	0.118	0.068	0.218	0.180
	1,000	None	0.996	0.996	0.998	0.998
		*Q*_1_	1.000	1.000	1.000	1.000
		*Q*_2_	1.000	1.000	1.000	1.000

**Table 3:** Numerical comparison of the Empirical Power of Adaptive Mantel test (i) with cross-validation vs. (ii) with Gamma approximation and cross-validation using the simulated data with *n* = 350, error standard deviation σ = 1 and $\text{b}\sim N\left(0,\sigma_{b}^{2}{\text{I}}_{p}\right),\sigma_{b}=0.50$.

			**Adaptive Mantel Test**			
			**With CV**		**With GA and CV**	
**Covariance structure**	**p**	**τ**	**GCV**	**LOOCV**	**GCV**	**LOOCV**
Compound symmetric	500	None	0.070	0.070	0.112	0.112
		*Q*_1_	0.196	0.152	0.230	0.192
		*Q*_2_	0.150	0.136	0.188	0.176
	1,000	None	0.998	0.998	0.998	0.998
		*Q*_1_	1.000	1.000	1.000	1.000
		*Q*_2_	1.000	1.000	1.000	1.000
Heteroskedastic	500	None	0.092	0.092	0.268	0.268
		*Q*_1_	0.084	0.092	0.266	0.268
		*Q*_2_	0.088	0.092	0.268	0.268
	1,000	None	0.996	0.996	0.996	0.996
		*Q*_1_	1.000	1.000	1.000	1.000
		*Q*_2_	1.000	1.000	1.000	1.000

Using the AdaMantCV algorithm and *p* = 1,000, Table 1 shows a decreasing Type I error rate when a threshold is imposed on the specification of the interval I as compared to using the unadjusted singular values. Also, using the median (*Q*_2_) as the threshold compared to the first quartile (*Q*_1_) leads to a lower proportion of incorrect rejections, regardless on whether GCV or LOOCV is utilized. It is important to note that in these cases, the empirical power are all approaching to 1 but the decreasing proportion of incorrect rejections for the AdaMantCV supports the proposed method in identifying the range of the ridge penalty parameters. In general, we observe an improved empirical power when thresholding is imposed.

**Figure 3:**
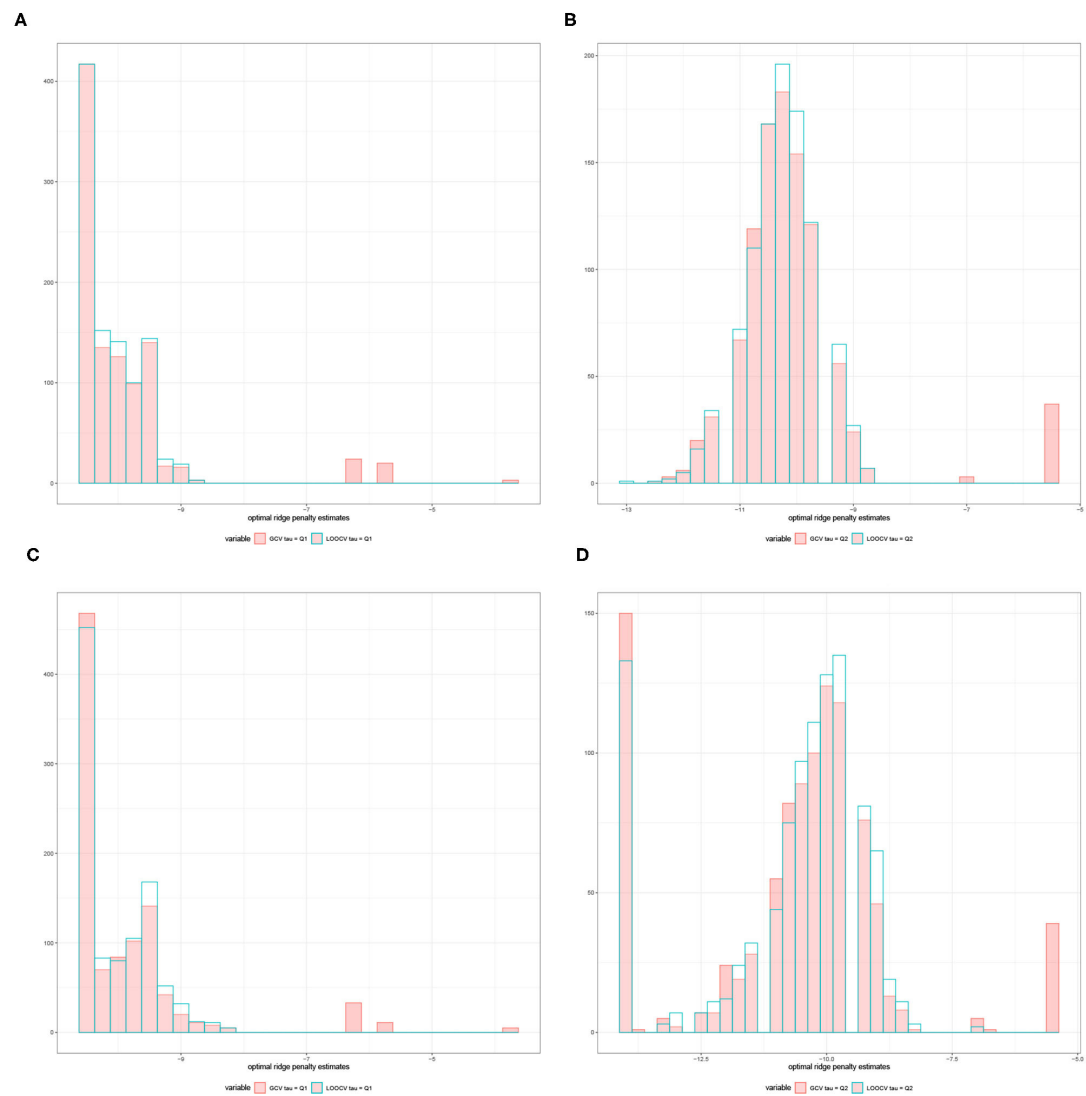
Histograms comparing the optimal ridge penalty parameter in a replication with 1,000 permutations using GCV vs. LOOCV when the threshold τ is the **(A)** first quartile and **(B)** median. The simulated design matrix **X** for **(A,B)** has a compound symmetric covariance structure with *n* = 350 and *p* = 1, 000, ***β*** = **0** and error standard deviation σ = 1. The simulated design matrix **X** for **(C,D)** has a compound symmetric covariance structure with *n* = 350 and *p* = 1, 000. The response is generated using fixed effects **β** = ξ**1**_*p*_, ξ = 3 and error standard deviation σ = 1.

For a given replication, the optimal ridge penalty is obtained for the original data and the *B* permutations of the data. A closer inspection of the distribution of ${\hat{\lambda }}^{\left(b\right)},b=0,1,\ldots ,B$-values are provided in Figure 3 for compound symmetric covariance, which was intensively studied in Kobak et al. (2020). In Figure 3A where the first quartile is used as a threshold, the average value of the ridge penalty parameter is −8.927 using GCV and −9.114 using LOOCV. Conversely, in Figure 3B where the median is the threshold τ, the average value of the ridge penalty parameter is −9.113 and −9.274 using GCV and LOOCV, respectively. When no thresholding is imposed, the average value of the ridge penalty parameter is the maximum allowable value of λ=1,000, using both GCV and LOOCV. These results were obtained from the setting where the simulated design matrix **X** has a compound symmetric covariance structure with *n* = 350 and *p* = 1, 000, ***β*** = **0** and error standard deviation σ = 1. In these numerical studies, we confirm that the optimal ridge penalty parameter is negative in some settings whenever a threshold is imposed. Furthermore, the results in Table 1 ensure us that using a negative value of the ridge penalty parameter still leads to a controlled Type I error rate for a given level α.

Consequently, Figure 3C displays the comparison of the optimal ridge penalty parameters across all permutations when τ is equal to the first quartile. The average value of the ridge penalty parameter is −9.867 using GCV and −10.023 using LOOCV in this setting. Meanwhile, when the median is utilized as threshold in Figure 3D, the average value of the ridge penalty parameter is −10.623 and −10.689 using GCV and LOOCV, respectively. When no thresholding is imposed, the average value of the ridge penalty parameter is 989.1 and 991.9, using GCV and LOOCV, respectively. Results were obtained using a simulated design matrix **X** with compound symmetric covariance structure, *n* = 350 and *p* = 1, 000. The response is generated using fixed effects **β** = ξ**1**_*p*_, ξ = 3 and σ = 1. We observe that some negative ridge penalty parameters have empirical power approaching 1. In these simulations, we were able to verify that this phenomenon can be observed in high-dimensional hypothesis testing where the empirical power approaches 1 as shown in Table 2.

In summary, our simulation studies provide evidence in favor of the proposed thresholding procedure discussed in Section 3.1. More importantly, Figure 3 illustrate the almost sure convergence results in Patil et al. (2021) wherein the distribution of the optimal ridge penalty estimates obtained using GCV and LOOCV coincide and exhibit the same pattern. This almost convergence result for GCV and LOOCV also applies to the Type I error and empirical power of the proposed association tests. Lastly, the Gamma approximation and cross-validation incorporated in the Adaptive Mantel test yields superior power while maintaining the proportion of false positives. This empirically justifies the use of Gamma distribution to approximate the null distribution of the ridge-penalized test statistic.

### 4.2. Application to Imaging Genetics Study

In this study, we consider data from 350 healthy college students from Beijing Normal University (BNU) who participated in an experiment involving visual working memory. For every trial, a 64-channel EEG was recorded at 1 kHz. These data constitute a subset of imaging, genetics, and behavioral data collected with the purpose of identifying neurological characteristics of brain connectivity that are significantly associated with both genetic variations and cognitive performance (Pluta et al., 2021).

**Figure 4:**
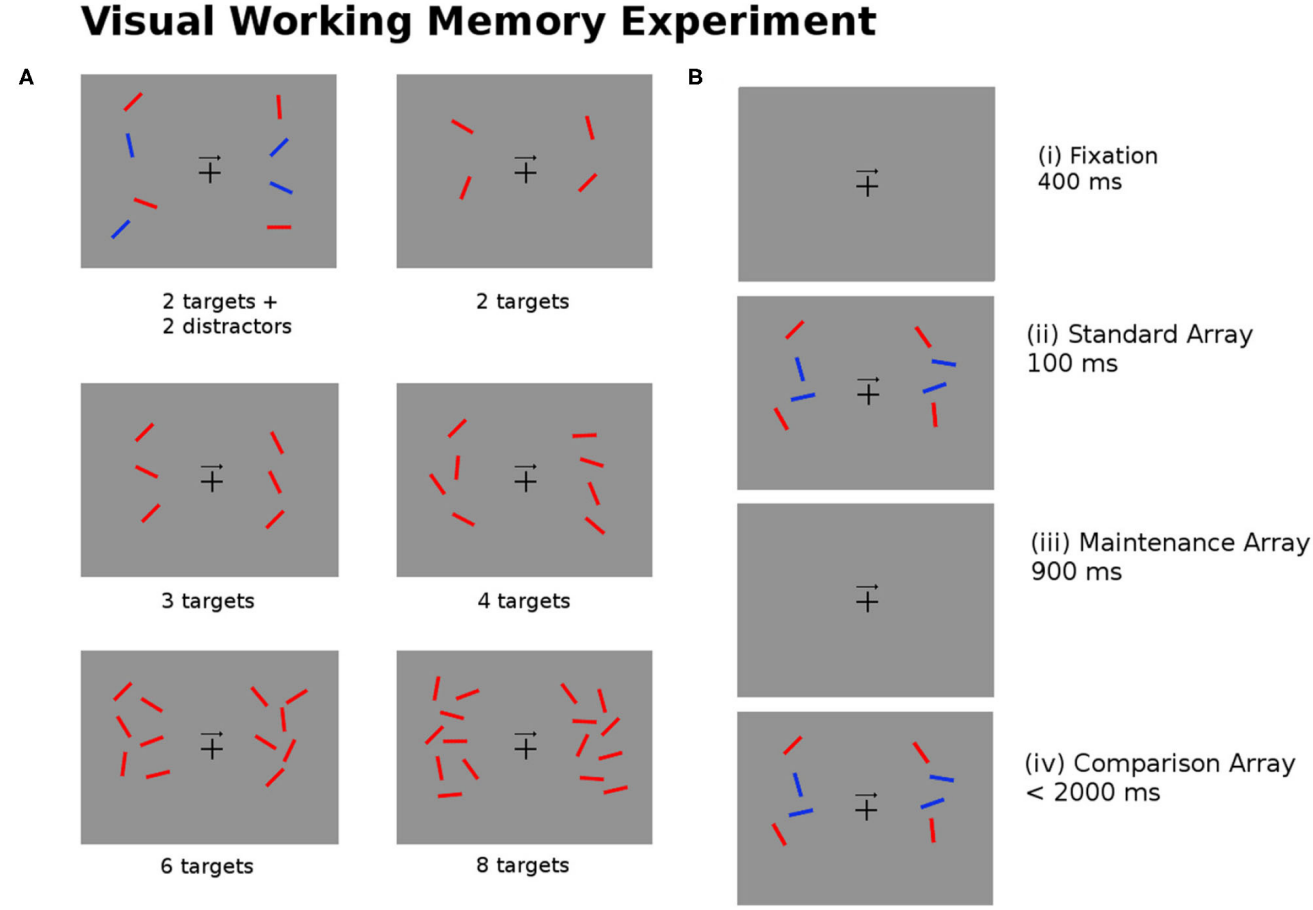
**(A)** The experimental task required subjects to recall the position of the red bars (targets) on the left or right side of the image as indicated by the arrow in the center of the image. The six images show examples of the test image for different numbers of targets and distractors (blue bars). **(B)** For every trial of the experiment the subjects are shown (i) a fixation image (400 ± 200 ms); (ii) the standard array to be memorized (100 ms); (iii) the maintenance image (900 ms); (iv) the comparison array, at which point subjects are asked to respond within 2,000 ms if the targets in the comparison arrays match or mismatch the standard array (Pluta et al., 2021).

The task for the participants involves remembering the position of the targets (red bars) on either the left or right side of the image as indicated by the arrow in the center of the image. There are six experimental conditions used for 2, 3, 4, 6, and 8 targets, and for 2 targets added with 2 blue distractors. Figure 4 describes the experimental task undertaken by the subjects.

The hypothesis being tested is whether the variation in a set of candidate SNPs is associated to the variation in EEG coherence. The main objective of association testing is to determine whether the heritability of EEG coherence in the delta (2–4 Hz), theta (4–8 Hz), alpha (8–12 Hz), beta (12–30 Hz), and gamma (30–50 Hz) bands is significantly greater than zero. In the initial study by Pluta et al. (2021), the matrix of explanatory variables corresponds to the genotype data including 484,496 autosomal SNPs which satisfies the minor allele frequency (MAF) and Hardy-Weinberg equilibrium (HWE) *p*-value quality control thresholds. In addition, the matrix of response variables includes 20,480 features which correspond to the pairwise coherence for 64 EEG channels and 5 frequency bands.

However, with only *n* = 350 subjects, we expect that association between the *q* = 20,480 channel-channel-frequency imaging features and *p* ≈ 500,000 SNPs to be challenging to detect. As mentioned in the previous sections, genetic variants are notorious for contributing fairly weak effects to disease risk due to heterogeneity, among others. Hence, we start with a candidate set of SNPs identified in a genome-wide association study on educational attainment by Okbay et al. (2016). Furthermore, we narrowed down the pairwise channel and frequency band features using the top results presented in Pluta et al. (2021). The resulting number of SNPs considered is *p* = 497 while the total number of brain connectivity features is 250. As mentioned previously, we will consider one response variable at a time, that is, the univariate scenario where *q* = 1.

#### 4.2.1. Optimal Ridge Penalty

**Figure 5:**
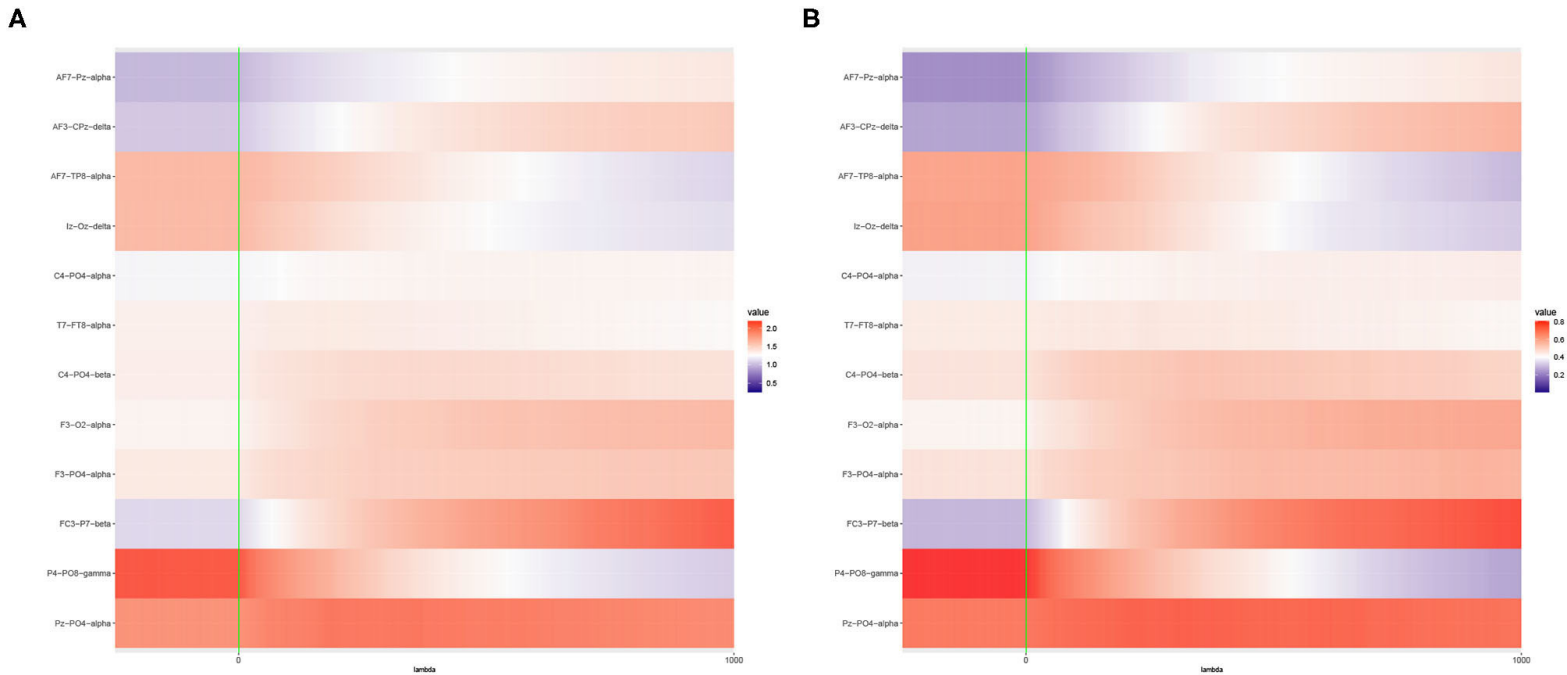
Heat maps of **(A)** Average of -log_10_ *p*-values and **(B)** Empirical Power of the Adaptive Mantel test using the imaging genetics data with *n* = 350 observations, *p* = 497 genetic features and coherence information from top channel-channel-band triples. The green vertical line corresponds to λ = 0.

**Figure 6:**
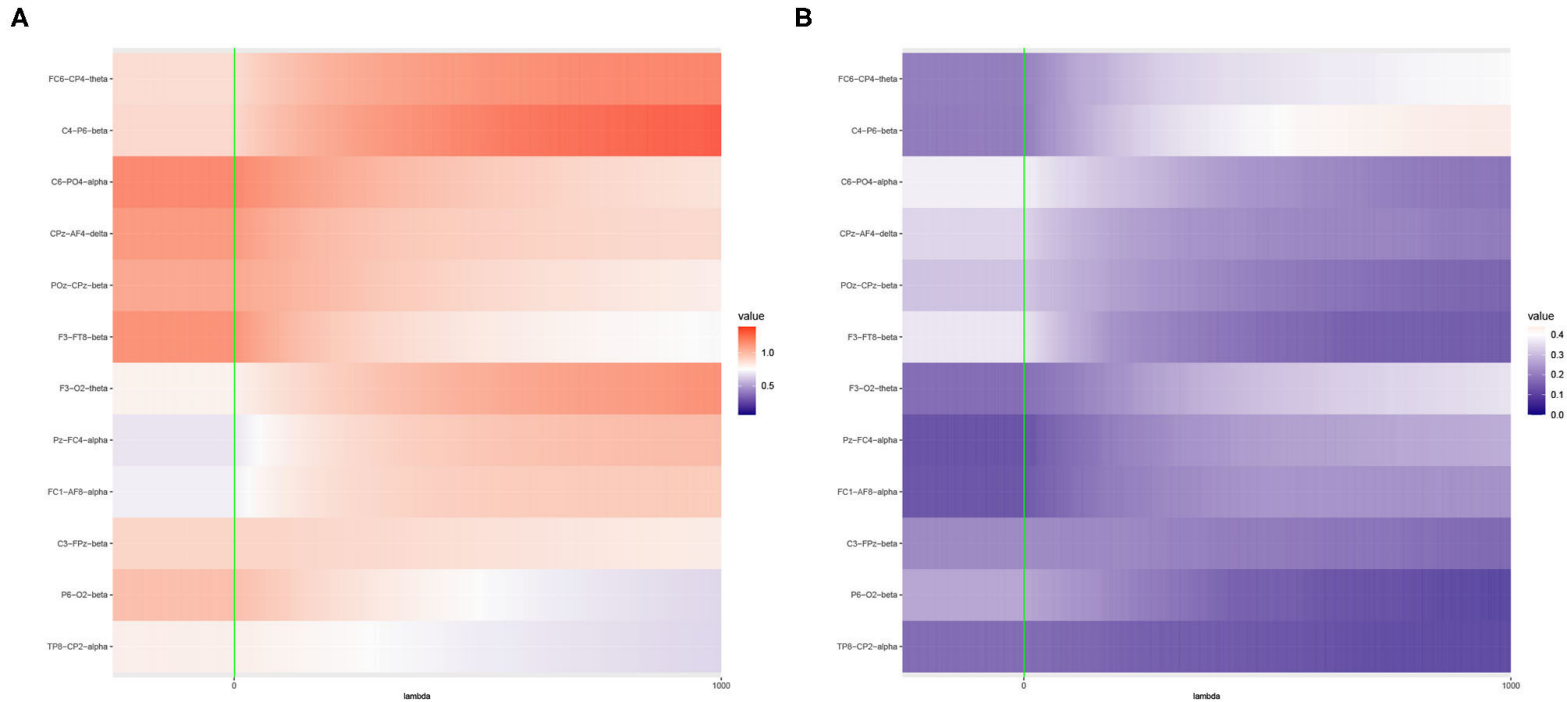
Heat maps of **(A)** Average of -log_10_ *p*-values and **(B)** Empirical Power of the Adaptive Mantel test using the imaging genetics data with *n* = 350 observations, *p* = 497 genetic features and coherence information from some channel-channel-band triples. The green vertical line corresponds to λ = 0.

The liver toxicity data set used in Section 2.4 is well-known for displaying strong signals and is typically used to demonstrate the predictive performance of newly developed vs. existing procedures. However, existing literature on imaging genetics studies suggest that the signals are weaker and sparse. In this section, we are interested in investigating the characteristics of the empirical power for several values of the ridge penalty parameter. For a fixed value of λ, we implement the empirical power calculation for the Adaptive Mantel test discussed in Section 2.4. The overall results are summarized in Figures 5, 6.

It is apparent that the brain imaging features considered in Figure 5 resulted to a higher empirical power compared to the features displayed in Figure 6. However, instead of just reporting channel-channel-band triples with high empirical power, we are interested in studying the underlying characteristics which prompted the results. In fact, the pattern of results observed in Figures 5, 6 can be categorized into three main groups of power trends. The cool to warm hue of the heat map indicate that there is an increasing trend in the empirical power as the value of ridge penalty parameter chosen increases. However, the warm to cool hue in the heat map describes the opposite trend. The third case corresponds to the almost constant hue for any ridge penalty included in the interval.

**Figure 7:**
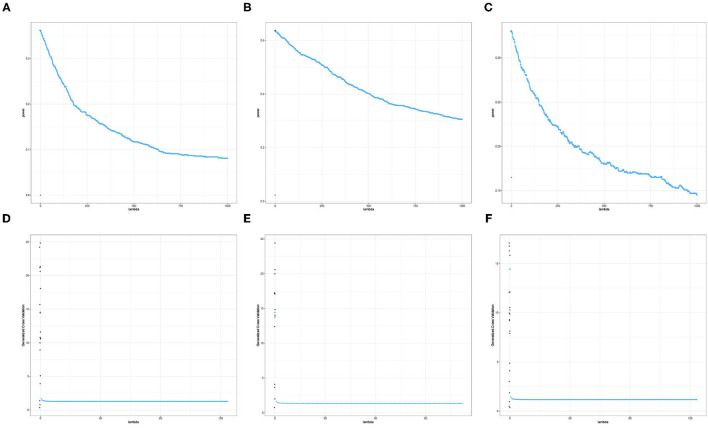
Empirical Power and GCV plots of the Adaptive Mantel test for the imaging genetics data with *n* = 350 observations, *p* = 497 genetic features and coherence information from selected channel-channel-band triples with decreasing power trend. **(A)** Power: F3-FT8-beta. **(B)** Power: Iz-Oz-delta. **(C)** Power: CPz-AF4-delta. **(D)** GCV: F3-FT8-beta. **(E)** GCV: Iz-Oz-delta. **(F)** GCV: CPz-AF4-delta.

**Figure 8:**
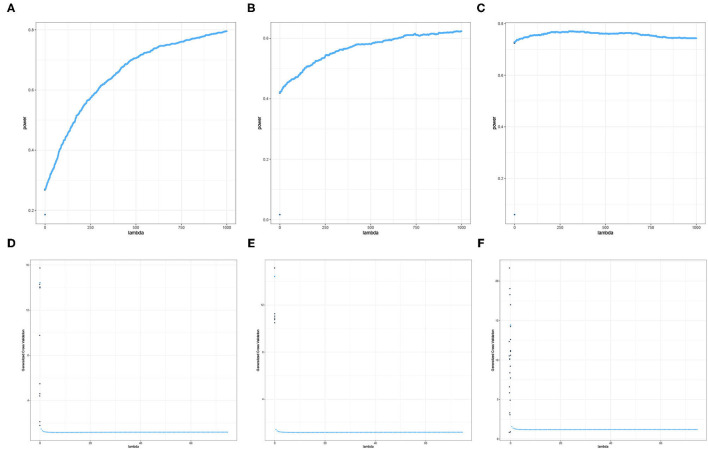
Empirical Power and GCV plots of the Adaptive Mantel test for the imaging genetics data with *n* = 350 observations, *p* = 497 genetic features and coherence information from selected channel-channel-band triples with increasing power trend. **(A)** Power: FC3-P7-beta. **(B)** Power: F3-O2-alpha. **(C)** Power: Pz-PO4-alpha. **(D)** GCV: FC3-P7-beta. **(E)** GCV: F3-O2-alpha. **(F)** GCV: Pz-PO4-alpha.

The results displayed in Figures 7, 8 illustrate clearly the increasing or decreasing power trends for some pairwise channel-band triples as compared to the subtle differences observed in Figures 5, 6. Among these overall cluster of results, we will explore further how the optimal ridge penalty parameter impacts the empirical power. Visually, we can deduce using Figures 7A–C that the optimal value of the ridge penalty parameter should be negative or close to zero to arrive at the highest value of empirical power. In contrast, Figures 8A,B suggest that the optimal ridge penalty should be chosen as high as possible, i.e., λ → ∞ to achieve empirical power approaching 1. Lastly, Figure 8C displays an almost horizontal trend where λ can be chosen anywhere in the interval and yield comparable empirical power with any other λ. The corresponding GCV plots for both Figures 7, 8 are provided to evaluate the pattern exhibited by the GCV for different values of λ. It is clear that when the ridge penalty parameter is positive, the value of the GCV is a smooth function. However, since this is the high-dimensional setting and multiple factors are at play simultaneously, it is not clear which dominating factor dictates the power trend and GCV plots displayed by the real data. We will probe into the theoretical justifications of this phenomena applied to high-dimensional or ultra-high-dimensional settings in future research.

#### 4.2.2. Adaptive Mantel Test With Cross-Validation

Among the 250 brain connectivity features studied, we identified five features which indicate that the variation in a set of candidate SNPs is associated to the variation in EEG coherence. Using the AdaMantCV and AdaMantGACV methods, we determine that the heritability of EEG coherence in the delta (2–4 Hz), alpha (8–12 Hz), and gamma (30–50 Hz) bands is significantly greater than zero. The *p*-values are presented in Table 4.

**Table 4:** Comparison of the *p*-values using AdaMantCV and AdaMantGACV methods where heritability of EEG coherence is significantly greater than zero.

		**Adaptive Mantel Test**			
		**With CV**		**With GA and CV**	
**Band**	**Channels**	**GCV**	**LOOCV**	**GCV**	**LOOCV**
Delta	FP1 - T8	0.016	0.015	0.012	0.012
Delta	CPz - F8	0.009	0.008	0.007	0.007
Delta	FC5 - O2	0.004	0.003	0.005	0.004
Alpha	F3 - FC1	0.037	0.038	0.006	0.007
Gamma	P4 - PO8	0.034	0.034	0.014	0.015

In addition, we were able to identify 21 other channel-channel-band triples which wherein the variation in a set of candidate SNPs is associated to the variation in EEG coherence using AdaMant with Gamma Approximation and cross-validation but not using AdaMantCV. The real data analysis results are aligned with the simulation studies wherein the Adaptive Mantel test with Gamma approximation and cross-validation have superior power while maintaining the proportion of false positives. Consequently, we have identified that there are more significant variations in the alpha and delta band frequencies using AdaMant with Gamma approximation and cross-validation. These results are consistent with the existing literature by Smit et al. (2005) where heritability is generally highest around the alpha peak frequency. According to Shaw (2003), the variation in the alpha rhythm has been posited to reflect individual differences in working memory, attentional demands and/or arousal, and also cognitive preparedness.

## 5. Discussion

For over several decades, ridge regression has proved to be a valuable tool for use by researchers and it has recently been intensively explored in the high-dimensional context to better understand more complicated models. Even though there are several available methods for choosing an optimal value of the ridge penalty parameter, the ultimate choice of λ for a specific application still remains to be unsolved. One contributing reason to this is the difficulty in characterizing the unknown data generating distribution, which usually influences the optimal level of regularization.

In this study, we examine the role that ridge penalization plays in hypothesis testing by conducting an empirical power study of an imaging genetics data set. Our results confirm that in high-dimensional settings, overfitting might provide higher power, in addition to good generalization in predictive problems. One noticeable difference is that while no penalty, i.e., λ = 0 often works well for predictions, it does not have any power in hypothesis testing, as typical test statistics reach their extreme values or they do not change over permutations when *p* > *n*. While an empirical study provides helpful guides for practical purposes, it is only the first step toward a more rigorous and holistic understanding of the broader scenarios. We are working on theoretical justifications and hope they will provide further insights to hypothesis testing problems into high-dimensional or ultra-high-dimensional settings.

We also propose a thresholding procedure to allow the set of candidate values of λ to include negative values and investigate how these negative penalty parameters can affect the empirical power of the Mantel Test. Furthermore, we extend the Adaptive Mantel Test (AdaMant) algorithm to incorporate Gamma approximation and the optimal selection of the ridge penalty parameter *via* generalized and leave-one-out cross-validation. We compare the resulting optimal choice between the two cross-validation procedures and note that they coincide. We also applied the proposed method to imaging genetics study of visual working memory measured by EEG coherence in healthy college students. Overall, we have encountered an interesting statistical phenomenon and gained some insights regarding ridge regression, especially as it applies to imaging genetics data.

## Data Availability Statement

The original contributions presented in the study are included in the article/supplementary materials, further inquiries can be directed to the corresponding author/s.

## Ethics Statement

Ethical review and approval was not required for the study on human participants in accordance with the local legislation and institutional requirements. The patients/participants provided their written informed consent to participate in this study.

## Author Contributions

IG and ZY drafting and refining the manuscript. ZY and HO critical reading of the manuscript. All authors contributed to the manuscript preparation of the article and approved the submitted version.

## Conflict of Interest

The authors declare that the research was conducted in the absence of any commercial or financial relationships that could be construed as a potential conflict of interest.

## Publisher's Note

All claims expressed in this article are solely those of the authors and do not necessarily represent those of their affiliated organizations, or those of the publisher, the editors and the reviewers. Any product that may be evaluated in this article, or claim that may be made by its manufacturer, is not guaranteed or endorsed by the publisher.
